# Transbrachial Access Site Complications in Endovascular Interventions: A Systematic Review of the Literature

**DOI:** 10.7759/cureus.25894

**Published:** 2022-06-13

**Authors:** Koushik Mantripragada, Kevin Abadi, Nikolas Echeverry, Sumedh Shah, Brian Snelling

**Affiliations:** 1 Department of Surgery, University of Maryland Medical Center, Baltimore, USA; 2 Emergency Medicine, Memorial Healthcare, Pembroke Pines, USA; 3 College of Medicine, Charles E. Schmidt College of Medicine at Florida Atlantic University, Boca Raton, USA; 4 Department of Neurosurgery, University of Miami, Miami, USA; 5 Neurosurgery, Boca Raton Regional Hospital, Boca Raton, USA

**Keywords:** transbrachial, complications, brachial artery, brachial, endovascular surgical repair

## Abstract

The transfemoral approach (TFA) or transradial approach (TRA) serves as the primary technique for most endovascular cases; however, the transbrachial (TBA) route is an alternative access site used when TFA and TRA are contraindicated. Although TBA has advantages over TRA, such as the ability to accommodate large guide catheters and devices, there is some apprehension in implementing TBA due to perceived access site complication rates. This article aims to glean the rate of access site complication from current literature.

Relevant studies were identified using the following search terms: ((access site complications) AND ((endovascular AND brachial) OR (percutaneous brachial access) OR (brachial))) OR (endovascular AND (percutaneous brachial access)); endovascular + brachial artery; endovascular + brachial artery + access site; and endovascular + brachial artery + access site complications. Articles published after 2008 addressing major complication rates from percutaneous TBA interventions were included.

Fifteen studies out of 992 total articles met the inclusion criteria. The major access site complication rate was 75/1,424 (5.27%). Patients who underwent hemostasis with a vascular closure device (VCD) had a major complication rate of 13/309 (4.21%) compared to a major complication rate of 65/1122 (5.79%) for patients who underwent hemostasis with manual compression (MC).

The major access site complication rate associated with TBA was 5.27%, which is relatively high compared to the complication rate in TFA or TRA. More prospective trials are needed to fully understand the access site complication rate in TBA interventions.

## Introduction and background

While the transfemoral approach (TFA) or transradial approach (TRA) serves as the primary access technique for a majority of endovascular cases, the transbrachial (TBA) route is an alternative access site occasionally used by interventionalists. TBA has been utilized in situations where TFA and TRA are contraindicated, such as small radial artery-to-sheath size ratio, unfavorable aortic arch anatomy, or prior femoral artery interventions precluding femoral access [1]. The brachial artery’s large luminal diameter also affords the ability to place large guide catheters and devices, compared to TRA. However, there is apprehension about implementing TBA, particularly in terms of perceived access site complication rates due to its role as the sole artery supplying the arm leading into its branches, the radial and ulnar artery [1].

Current literature on brachial artery access complication rates has been limited to single-center retrospective studies with a paucity of prospective data. Thus, there has been controversy in establishing optimal technique guidelines [1]. Furthermore, there have been no standard characterizations of brachial access site complications in the literature, which has prevented the formal evaluation of TBA as an alternative access strategy. This is likely because, compared to access complications in the current interventional data, the TBA data is inconsistent in defining major access site complications [2]. Therefore, we sought to perform a systematic review of the literature to analyze access site complication rates and distinguish between major and minor access site complications in endovascular interventions utilizing TBA.

## Review

Methods

Search and Information Sources

This systematic review was performed in accordance with the PRISMA guidelines [3]. A search of the PubMed (MEDLINE) database was performed to locate relevant articles. Relevant studies were identified using the following search terms: ((access site complications) AND ((endovascular AND brachial) OR (percutaneous brachial access) OR (brachial))) OR (endovascular AND (percutaneous brachial access)); endovascular + brachial artery; endovascular + brachial artery + access site; and endovascular + brachial artery + access site complications. Articles considered for review were those published from 2008 to 2020, published in English, and utilized humans as participants. This date range was selected from our prior experience in TFA literature.

Eligibility Criteria and Study Selection

Articles included in this review must mention major access site complications in endovascular procedures utilizing percutaneous TBA access. Case reports and articles utilizing the TBA access for arteriovenous fistula creation or trauma patients were excluded. Additionally, articles that utilized surgical cutdown were excluded because surgical cutdown has been shown to have significantly fewer brachial artery access site complications compared to percutaneous access [4]. Studies were not excluded based on patient age. Major access site complications must meet any of the following criteria: bleeding requiring transfusion, require surgical/interventional radiology reintervention, or further intervention. This definition of major access sites was adapted from the ECLIPSE trial and Bhatty et al. [5,6]. Examples include hematoma requiring transfusion or surgical repair, pseudoaneurysm requiring surgical intervention, development of compartment syndrome requiring surgical intervention, and occlusion requiring thrombectomy.

Data Collection Process

**Figure 1:**
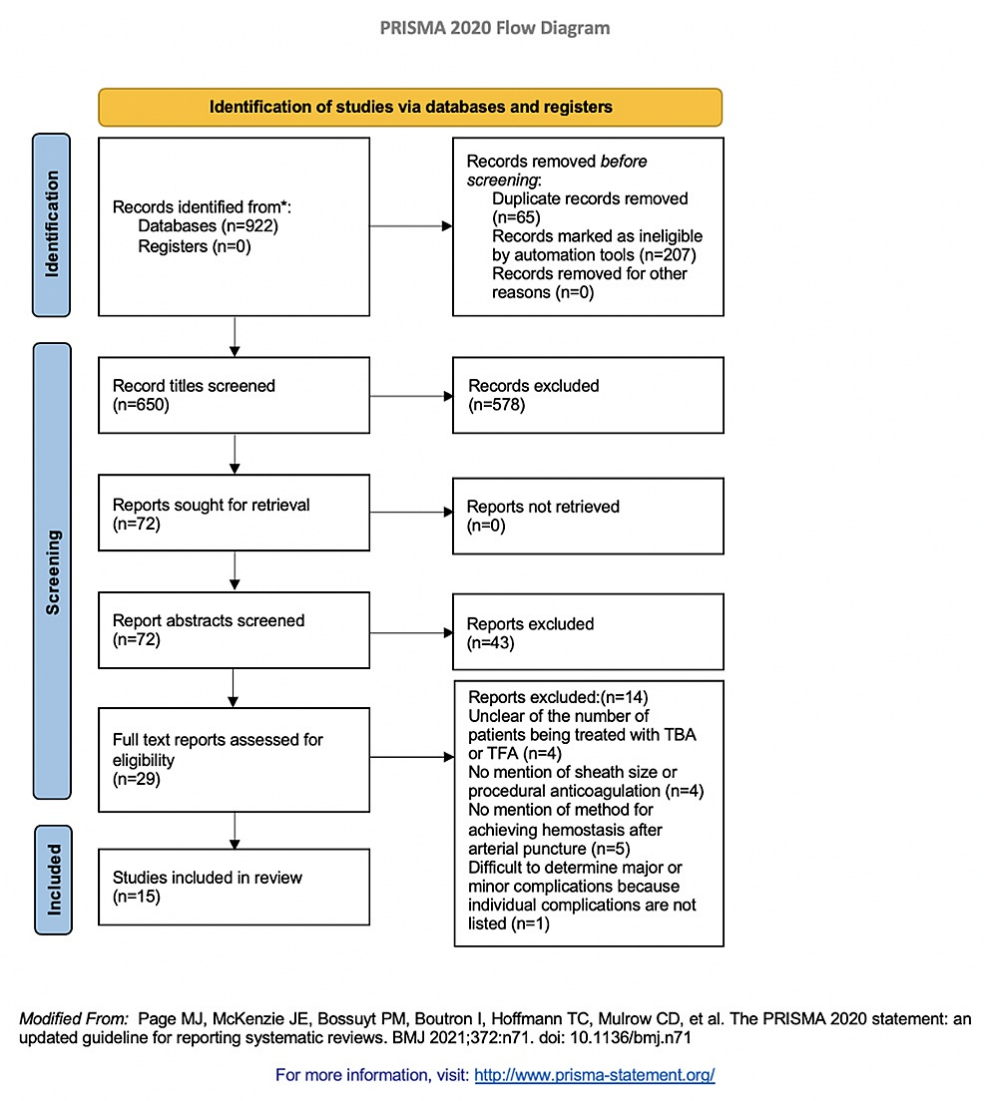
PRISMA Flowchart

The initial search identified 992 articles that were then compiled into a single database, after which irrelevant and duplicate articles were removed, resulting in 650 total articles. After preliminary screening, 72 articles remained, which were then assessed for eligibility, resulting in 29 articles included in the qualitative synthesis. The 29 articles were critically evaluated by three of the authors (KM, KA, and NE), and data regarding brachial access site complications were compiled into a data bank. A total of 14 papers were excluded upon complete analysis [7-20]. The articles excluded are outlined in Table 1. After a thorough systematic review, 15 articles were analyzed for access site complication rates (Figure 1) [2,21-34].

**Table 1:** Excluded Articles

Author	Reason for Exclusion
Bertoglio et al. [7]	Unclear number of patients treated with TBA
Fioole et al. [8]	Unclear number of patients treated with TBA
Liu et al. [9]	Unclear number of patients treated with TBA
Kim et al. [10]	Unclear whether complications are from TBA or TFA
Franz et al. [11]	No mention of sheath size or procedural anticoagulation
Moise et al. [12]	No mention of sheath size or procedural anticoagulation
Wu et al. [13]	No mention of sheath size or procedural anticoagulation
Wu et al. [14]	No mention of sheath size or procedural anticoagulation
Onishi et al. [15]	No mention of the method for achieving hemostasis after arterial puncture
Kim et al. [16]	No mention of the method for achieving hemostasis after arterial puncture
Parviz et al. [17]	No mention of the method for achieving hemostasis after arterial puncture
Ahmed et al. [18]	No mention of the method for achieving hemostasis after arterial puncture
Lee et al. [19]	No mention of the method for achieving hemostasis after arterial puncture
Mirza et al. [20]	Difficult to determine major or minor complications because individual complications are not listed

Results

Individual Study Characteristics

The methodology for each article is summarized in Table 2. The studies differ in terms of the endovascular procedure performed, anticoagulation status, use of vascular closure device (VCD) or manual compression (MC) after the procedure, and sheath size.

**Table 2:** Summary of Clinical Trial Methodology ^1^Vascular closure device or manual compression ACT: activated clotting time

Authors	Intervention	Sheath Size	Procedural Anticoagulation	VCD or MC^1^
Alvarez-Tostado et al. [21]	Diagnostic and therapeutic endovascular occlusion	4F – 9F	Heparin, ACT ≥ 300 seconds if endovascular abdominal or thoracic aneurysm	MC
Madden et al. [2]	Diagnostic and therapeutic endovascular interventions	5F – 7F	Systemic heparinization after access to maintain ACT > 200 seconds	MC
Meertens et al. [22]	Endovascular interventions on the thoracic and abdominal aorta	6F – 8F	5,000 IU heparin ± 2,500 IU heparin for ACT > 250 seconds	VCD and MC
Lupattelli et al. [23]	Endovascular treatment of critical limb ischemia	6F	5,000 IU with additional IU to maintain ACT > 250 seconds	VCD and MC
Stavroulakis et al. [24]	Iliac endovascular interventions	4F – 7F	ASA at baseline, heparin 5,000 IU after sheath insertion	MC
Stavroulakis et al. [25]	Endovascular treatment of peripheral arterial disease	5F or 6F	Heparin 5,000 IU after sheath insertion	MC
Treitl et al. [26]	Endovascular treatment of peripheral arterial disease	4F – 7F	Low-molecular-weight heparin (LMWH) SQ; patients had INR ≤ 1.5; patients who were not taking or stopped aspirin therapy prior to the procedure received an intravenous 500 mg bolus; if the endovascular procedure was to include any stenting or the use of drug-eluting devices, the patients received a 300 mg oral loading dose of clopidogrel on the day of the procedure	MC
Wei et al. [27]	Endovascular treatment of type B aortic dissection	6F	DAPT (ASA 100 mg QD, clopidogrel 75 mg QD), interventional procedure bolus of weight-based heparin	VCD and MC
Bechara et al. [28]	Endovascular treatment of recanalization of flush iliac artery occlusion	6F	Fully heparinized	MC
Millon et al. [29]	Endovascular treatment of TASC C-D aortoiliac occlusion in case of failed femoral access	5F	IV heparin 50 UI/kg and ASA 100 mg at the beginning of the procedure	MC
Puggioni et al. [30]	PTA stenting target vessels	6F	Systemic heparin 5,000 IU	VCD
Anton et al. [31]	Endovascular treatment of visceral artery aneurysm	5F	5,000 units of heparin in elective cases; in emergent cases presenting with bleeding, no anticoagulation was administered	VCD
van Dijk et al. [32]	Mesenteric arterial procedures	4F – 7F	5,000 IU intra-arterially^3^	MC
Troisi et al. [33]	Endovascular treatment of atherosclerotic iliac artery disease	4F	If thrombotic occlusion was present, an intra-arterial catheter was placed to deliver urokinase (80,000-100,000 IU/hour) and heparin (800-1,000 U/hour) to reach an activated partial thromboplastin time	MC
Varcoe et al. [34]	Endovascular reconstruction of the occluded aortoiliac segment	5F – 6F	IV heparin 5,000 U	MC

Data Analysis

Access site complications are outlined in Table 3 and ranged from 2.5% to 25%. There was inconsistency between studies in defining major access site complications; thus, our definition was used to calculate the total major access site complications.

**Table 3:** Overall Access Site Complication Rates ^1^A subset of patients had planned surgical cutdown; however, the study separated these patients from patients undergoing percutaneous access. ^2^Patients had pseudoaneurysm but were treated conservatively with ultrasound compression.

Author	Serious Adverse Event	Non-major Adverse Event
Alvarez-Tostado et al. [21]	13/289 (4.5%)	8/289 (2.8%)
Madden et al.^1^ [2]	15/142 (11%)	N/A
Meertens et al. [22]	2/19 (11%)	4/19 (21%)
Lupattelli et al. [23]	9/249 (3.6%)	19/249 (7.6%)
Stavroulakis et al. [24]	13/201 (6.5%)	12/201 (6%)
Stavroulakis et al. [25]	1/28 (3.6%)	N/A
Treitl et al.^2^ [26]	4/150 (2.7%)	21/150 (14%)
Wei et al. [27]	3/157 (2.5%)	29/157 (19%)
Bechara et al. [28]	1/10 (10%)	1/10 (10%)
Millon et al. [29]	2/39 (5.1%)	N/A
Puggioni et al. [30]	1/29 (3.4%)	1/29 (3.4%)
Anton et al. [31]	1/5 (20%)	N/A
van Dijk et al. [32]	8/52 (15%)	13/52 (25%)
Troisi et al. [33]	1/46 (2.2%)	N/A
Varcoe et al. [34]	1/8 (13%)	N/A

The access site complication rate was calculated by dividing the total number of access site complications by the total number of participants undergoing brachial access site interventions. The access site complication rate from pooled data was 183/1424 (12.9%), with a subgroup analysis revealing a major access site complication rate of 75/1424 (5.27%). With further subgroup analysis, a major complication rate was gleaned for patients undergoing hemostasis with MC versus VCD as outlined in Table 4.

**Table 4:** Complication Rates in VCD versus MC Patients

Method of Hemostasis	Serious Adverse Event	Non-major Adverse Event
VCD	13/309 (4.2%)	25/304 (8.2%)
MC	65/1122 (5.8%)	83/859 (9.6%)

Patients who underwent hemostasis with a VCD had a major complication rate of 13/309 (4.21%) compared to a major complication rate of 65/1,122 (5.79%) for patients who underwent hemostasis with MC.

Discussion

The brachial artery is occasionally used for access when there is an anatomic distortion of the femoral artery, femoral occlusive disease, and absent femoral pulses or when radial access is not feasible [21]. However, the brachial artery is an end artery and therefore could result in the loss of blood supply to the arm and hand with occlusion [26]. Prior studies have cited a complication rate of 6%-11% with endovascular brachial artery interventions [2,21,23]. This is compared to a complication rate of 1.4%-3.7% with endovascular femoral artery interventions and 1.9% with endovascular radial artery interventions [2,35].

The current data for TBA interventions is largely retrospective, and these studies may underreport major complications by virtue of failing to prospectively track complications. Moreover, there is no standard definition of major access site complications in these retrospective studies. For example, Mirza et al. defined major complications as including complications such as brachial thrombosis, limb ischemia, and complications requiring additional surgery [20]. Not included in this definition are complications that required other interventions such as a hematoma requiring transfusion.

Therefore, we defined major access site complications as complications requiring further intervention such as hematoma or bleeding requiring transfusion or complications requiring further surgical or endovascular interventions. This definition is adapted from the ECLIPSE trial and Bhatty et al. [5,6].

Our review found a major access site complication rate of 5.27% for patients undergoing TBA for endovascular interventions. The access site complication rate may be greater than the reported figure because the included trials were primarily retrospective. We were able to stratify the complication rate based on the method of hemostasis. Patients who underwent hemostasis with MC had a higher complication rate than patients who underwent hemostasis with VCD. Although the study of Mirza et al. was excluded from this systematic review, they have outlined that the differences between complication rates in the VCD and MC groups were not significantly different [20].

We have not attempted to stratify the complication rate based on sheath size because there are few articles that list the complication with associated sheath size. Out of the 29 articles reviewed for this systematic review, there were three articles that stratified complications based on sheath size [2,16,26]. However, Stavroulakis et al. posit, through a regression analysis, that arterial sheath size did not seem to have an influence on the access site complications rate [24]. A better attempt to stratify complication rates based on sheath size would be possible if future trials leave less ambiguity related to access site complications and associated sheath size.

There is a paucity of prospective literature regarding access site complications utilizing the brachial artery for access in endovascular interventions. Therefore, this study has utilized the available literature to glean a better understanding of the complication rates of TBA interventions. Without prospective studies, it is difficult to glean a true access site complication rate.

Several studies were excluded due to ambiguity related to reporting access site complications. Some of these studies attempted to gain access through multiple access sites but failed to specify which access site the complications are associated with. Similarly, the method of hemostasis was not mentioned in other articles. In some articles, there is no mention of sheath size or procedural anticoagulation. A better understanding of TBA access site complications would be possible if there is less ambiguity in reporting these complications.

## Conclusions

There is a need for better, more precise language in analysis for access site complications utilizing the brachial artery for endovascular interventions, especially neuromuscular procedures. However, we maintain that the brachial access complication rate appears to be higher than those of TFA or TRA. We suggest further investigation into the access site complication rate in endovascular interventions utilizing the brachial artery.
